# Obesity-Induced Changes in Bone Marrow Homeostasis

**DOI:** 10.3389/fendo.2020.00294

**Published:** 2020-05-12

**Authors:** Andrea Benova, Michaela Tencerova

**Affiliations:** Molecular Physiology of Bone, Institute of Physiology of the Czech Academy of Sciences, Prague, Czechia

**Keywords:** bone marrow microenvironment, bone marrow mesenchymal stem cells, hematopoietic stem cells, immune responses, obesity, life-style interventions

## Abstract

Obesity is characterized by low-grade inflammation, which is accompanied by increased accumulation of immune cells in peripheral tissues including adipose tissue (AT), skeletal muscle, liver and pancreas, thereby impairing their primary metabolic functions in the regulation of glucose homeostasis. Obesity has also shown to have a detrimental effect on bone homeostasis by altering bone marrow and hematopoietic stem cell differentiation and thus impairing bone integrity and immune cell properties. The origin of immune cells arises in the bone marrow, which has been shown to be affected with the obesogenic condition via increased cellularity and shifting differentiation and function of hematopoietic and bone marrow mesenchymal stem cells in favor of myeloid progenitors and increased bone marrow adiposity. These obesity-induced changes in the bone marrow microenvironment lead to dramatic bone marrow remodeling and compromising immune cell functions, which in turn affect systemic inflammatory conditions and regulation of whole-body metabolism. However, there is limited information on the inflammatory secretory factors creating the bone marrow microenvironment and how these factors changed during metabolic complications. This review summarizes recent findings on inflammatory and cellular changes in the bone marrow in relation to obesity and further discuss whether dietary intervention or physical activity may have beneficial effects on the bone marrow microenvironment and whole-body metabolism.

## Introduction

Bone marrow (BM) is a soft tissue localized inside of the bones and represents ~5% of total body mass in healthy individuals (1). BM is primary recognized as a hematopoietic organ supporting the production of new blood cells (2). However, it has also a mechanical and immune function as it comprises bone marrow mesenchymal stem cells (BMSCs), important building blocks for bone formation, and hematopoietic stem cells (HSCs) responsible for producing several types of immune cells crucial for immune responses (3, 4). While BMSCs promote bone tissue regeneration by osteoblast differentiation and neo-vascularization thereby supporting growth of a new tissue, HSCs are quiescent cells (5, 6). However, in response to external cues HSCs can mobilize to the site of inflammation, A majority of HSCs reside in BM and 0.01% of them can migrate into circulation (7). Circulating HSCs in peripheral blood are attracted by several biochemical factors and cytokines including SCF, CXCL12, or IL-8 (8, 9). The initiation of hematopoiesis starts in the fetal liver, where HSCs proliferate and then migrate to BM. Later during adulthood HSCs continuously migrate from BM to peripheral blood, which maintains steady hematopoiesis (10). In the process of HSC migration from BM, stem cells leave proliferative niches and migrate to more oxygenated and vascularized regions in BM (11). In cases of stress, injury or pharmacological intervention, alterations in HSC niche formation and interaction with BMSCs lead to HSC mobilization and egress. These processes are affected by the metabolic status of an organism, which is altered by caloric restriction, obesity and type 2 diabetes (12). However, it is not well-documented how the composition of BM, interaction between HSCs and BMSCs, and the inflammatory status in this organ are affected in metabolic complications.

Thus, the purpose of this review is to give an overview of the latest literature on inflammatory changes in the BM microenvironment in relation to bone homeostasis. Also, we will discuss how BM composition and secretory function change in different metabolic states and whether dietary intervention or physical activity may have beneficial effects on the BM microenvironment and whole-body metabolism.

## Bone Marrow as an Immune and Endocrine Organ

**Figure 1:**
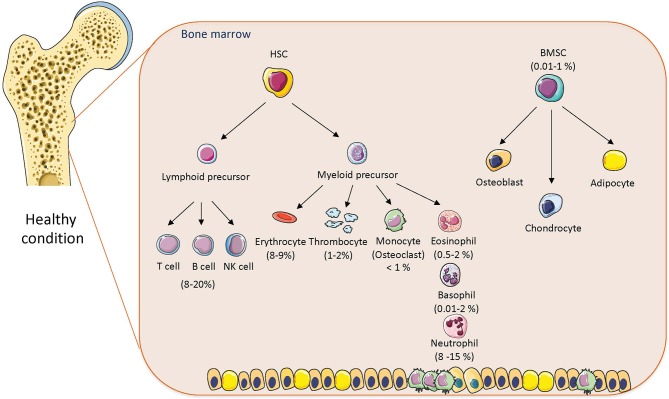
The cellular composition of bone marrow in healthy conditions. The composition of hematopoietic stem cells (HSCs) and bone marrow mesenchymal stem cells (BMSCs) with percentage in bone marrow in normal physiological condition. Cell animations were adapted from SERVIER Medical Art; https://smart.servier.com to create the figure.

BM is a heterogeneous immune organ, which consists of various cell types with different immune functions, including HSCs (myeloid and lymphoid precursors), which are important for immune cell production and BMSCs with immunosuppressive properties (3, 4, 13). It has been reported that 8–20% of BM mononuclear cells belong to lymphocyte lineage (T cells, B cells, Tregs) (14, 15) and approximately 1% represent plasma cells contributing to antibody production (16). Also located in the BM are natural killer T cells (NKT) (cca 0.4–4%) (17), dendritic cells (1–2%) (18), myeloid progenitor cells (giving rise to osteoclasts), megakaryocytes important for platelets (thrombocytes) production via thrombopoietin (1%) (19), neutrophils (8–15%), eosinophils (0.5–2%), and basophils (0.01–2%) (Figure 1). Importantly, BM represents a major reservoir of neutrophils and provides migration of these cells into circulation as a first host defense in response to infection and stress (20). Neutrophils are also cleared in BM; once they are senescent, they are phagocytosed by stromal BM macrophages (21). Thus, BM is a home of immune and progenitor cells, whose composition can be changed with age, metabolic status, or inflammatory condition.

Further, BM is well-vascularized with blood vessels and sinusoids, which create a barrier between BM and peripheral circulation (22, 23). This microvasculature allows a release of proliferating progenitor cells and secreted molecules from BM into blood stream in order to reach peripheral tissues depends on the stimulatory signals or physiological condition, which modulate a local microenvironment of the target tissue (24).

Early in life, many bones contain red BM with a high hematopoietic activity, which decrease and turn red BM into yellow “fatty” BM with aging (25). In adults, there are few bones with red BM (e.g., sternum, vertebrae, ribs, or pelvic bone) contributing to hematopoiesis (26). Thus, the bone homeostasis at different body sites is affected by BM composition of HSCs and BMSCs, which contribute to bone integrity, and mechanical and immune properties (4, 27). The crosstalk between these cells activate several processes, including proliferation, migration, and differentiation of stem cells, which are accompanied with production of various bioactive molecules creating the BM microenvironment (3, 4, 25). The maintenance of this microenvironment is important for healthy cell development, immune system function and metabolism.

## Intrinsic Regulators of Hematopoietic Stem Cell and Bone Marrow Mesenchymal Stem Cell Differentiation

HSCs and BMSCs represent multipotent stem cells, which can differentiate into different cell types based on the regulation via intrinsic (e.g., transcription factors and cofactors, posttranscriptional and posttranslational modifications) and extrinsic factors (e.g., secretory molecules, BM microenvironment, metabolic cues) (28). HSC differentiation is coordinated by transcription factors such as c-Myc, PU.1/Spi-1, GATA1-3, TNFβ, EGR1, BMI1, Gfi1, FoxO3, and others (29). c-Myc, for example, regulates the balance between HSC self-renewal and differentiation (30). PU.1/Spi-1 is involved in myeloid lineage determination via regulation of target genes, including granulocyte colony-stimulating factor receptor (31), granulocyte-macrophage colony-stimulating factor receptor (32) and macrophage colony-stimulating factor receptor (33). Some studies showed that PU.1/Spi-1 expression can direct stem cell differentiation to myeloid lineage if Notch signaling is reduced [reviewed in Rothenberg et al. (34)]. Another regulatory molecule of HSC differentiation is Ikaros, which displays a crucial function as a transcription activator promoting lymphocyte differentiation. Impairment of this protein leads to hypoplasia, absence of secondary lymphoid organs or absence of B- and T- cell precursors (35). Basic leucine zipper transcription factor, ATF-like (BATF) is an important factor promoting lymphoid lineage differentiation (36), while TNFβ serves as a negative regulator of HSC self-renewal (37). Further, the HSC cell fate determination is regulated by GATA1-3, zinc finger transcription factors, which coordinate development of diverse hematopoietic lineages (38), and B lymphoma Mo-MLV insertion region 1 homolog (BMI1), which is important for the multilineage potential of HSCs and their replating capacity (39, 40). Lee et al. recently identified a role of a transcriptional repressor, known as Gfi1 in the regulation of HSC quiescence and self-renewal, which is modulated by metabolic status (i.e., upregulated with obesity and decreased with weight loss) (41). Besides transcriptional regulation, HSC renewal and differentiation are under control of posttranslational modifications, including DNA methylation, acetylation, or ubiquitination, which can be modulated by aging or metabolic diseases (42). Recent findings documented that increases in H4K16Ac levels results in inhibition of Cdc42, which leads to restoration of the B cell lineage output in aged HSCs (43). Further, G9a/GLP methyltransferase is responsible for increased levels of H3K9me2 pattern associated with HSC lineage commitment. On the other hand, inhibition of G9a/GLP decrease differential potential of stem cells and improves HSC maintenance (44). Additionally, methylation by DNA methyltransferase 1 (DNMT1) permits efficient hematopoietic differentiation (45). All above-mentioned transcription factors and posttranslational modifications are only part of the HSC regulatory network, which shows together the complexity of stem cell differentiation process.

Differentiation of BMSCs toward osteoblasts and adipocytes is regulated by specific transcription factors: Runt-related transcription factor 2 (Runx2) (46), osterix (47), GATA2 (48, 49) (responsible for osteoblast lineage determination), and peroxisome proliferated-activated receptor gamma (PPARγ) (50), CAAT enhancer binding protein (C/EBP) family (4) (responsible for adipocyte lineage determination). The activation of these transcription factors can be controlled by Wnt signaling, transforming growth factor β_1_ (TGF-β_1_) and bone morphogenic proteins (BMPs) [reviewed in Tencerova and Kassem (4)]. The regulation of BMSC differentiation is also accompanied by epigenetic modifications. For example, histone deacetylation in genes involved in transcriptional regulation, cellular survival, growth and proliferation of BMSCs. Increased acetylation during osteoblast differentiation results in increased expression of Runx2, BMP-2, osterix and osteopontin (OPN), which are important for osteoblast maturation (51, 52). A recent study by Addison et al. identified Zfp521 as a key regulator of lineage specification in progenitor cells, regulating BMP-induced MSC differentiation coupled with histone modification at Zfp423 promoter (53).

These data demonstrate that HSC and BMSC differentiation are complex processes under the control of specific transcription factors, whose activity is further epigenetically modulated. These intrinsic factors contribute to the regulation of the BM homeostasis and are changed by obesity and dietary interventions. Table 1 summarizes key factors and determinants regulating HSC and BMSC differentiation and associated signaling pathways.

**Table 1:** Transcription factors and determinants of HSC and BMSC differentiation.

**Intrinsic regulators**	**Cell type**	**Function**	**Obesity**	**Life-style interventions**
BATF	HSC	Transcription factor regulating lymphoid differentiation (36)	–	–
BMI1	HSC	Transcription factor regulating multilineage potential of HSCs (39, 40)	–	–
TNFβ	HSC	Negative regulator of HSC self-renewal (37)	–	–
c-Myc	HSC	Transcription factor regulating balance between HSC self-renewal and differentiation (15545632)	–	–
Pu.1/Spi-1	HSC	Transcription factor regulating myeloid lineage differentiation (31)	↑ (54, 55)	–
Ikaros	HSC	Transcription activator of lymphoid differentiation (35)	–	–
Notch	HSC	Signaling molecule enhancing self-renewal and regenerative capacity of HSCs (56)	–	–
GATA1-3	HSC	Transcription factors regulating HSC lineage determination (38)	↑ GATA 3 (57)	–
Gfi1	HSC	Transcription factor regulating HSC quiescence and self-renewal (58)	↑ (59)	↓ Weight loss (59)
DNMT1	BMSC	DNA methyltransferase promoting HSC differentiation to myeloid lineage (45)	–	–
Runx2	BMSC	Transcription factor promoting osteoblast differentiation (46)	= (60)	↑ Vibration (61)
Osterix	BMSC	Transcription factor promoting osteoblast differentiation (47)	↓ (62)	↓ Low magnitude high frequency vibration (63)
PPARγ	BMSC	Transcription factor regulating adipogenesis (50)	↑ (60)	↑ Low magnitude height frequency vibration (64)
GATA2	BMSC	Transcription factor regulating adipogenesis and osteogenesis (48, 49)	–	–
C/EBP	BMSC	Transcription factor regulating adipogenesis (4)	↑ (65)	↑ Low magnitude height frequency vibration (64)
TGF-β_1_	BMSC	Negative regulator of adipogenesis (4)	↓ (66)	–
BMP-2	BMSC	Positive regulator of osteoblast differentiation (51)	↓ (67)	= Calorie restriction (68)
Zfp521	BMSC	Regulator supporting osteoblast differentiation (53)	–	–

## Secretory Factors of Bone Marrow Mesenchymal Stem Cells Affecting Bone Homeostasis and Immune Cell Properties

BMSCs represent around 0.01–0.1% of total BM cells in adults and are capable of differentiating into different cell types such as osteoblasts (bone formation), adipocytes (adipose tissue formation) or chondrocytes (cartilage formation), all of which are important for maintaining of bone homeostasis (4, 13). BMSCs are also known for their immunosuppressive properties as they express human leucocyte antigen (HLA) class I and costimulatory molecules CD80, CD86, or CD40 important for regulation of T cell proliferation and activation (69, 70). Recent findings suggest that BMSCs mediate their immunoregulatory function via cell-cell interactions and secretion of soluble molecules (70, 71). The BMSC secretory profile of prostaglandin E_2_ (PGE_2_), hepatocyte growth factor (HGF), transforming growth factor (TGF)-β_1_ and indoleamine 2,3-dioxygenase (IDO) show profound immunosuppressive properties inhibiting T cell activation and proliferation without affecting expression of early activation markers such as CD25 and CD69 (72). Indeed, BMSCs can modulate function of several immune cells without being recognized by immune cells. BMSCs inhibit proliferation and antibody production of B cells (73), differentiation of HSC progenitors into dendritic cells (74), and they promote anti-inflammatory cytokine production of myeloid cells while inhibiting the cytotoxic activity of NKT cells (75–77). Although there is limited information on the exact mechanism of BMSC immunoregulation in relation to immune cell interaction, they represent an important tool in stem cell therapy. BMSCs have been used in several clinical trials for tissue regeneration and healing (78–81).

The secretory profile of BMSCs may differ depend on developmental, immune or metabolic challenges they are exposed to. A brief overview of BMSC secretory factors is listed in Table 2, describing their functions and changes in metabolic complications and life-style interventions. BMSCs secrete IL-7, which is important for early B cell development (83, 84), IL-15 for T cell homeostasis (85) and IL-21 for maturation of NKT cells (87). Further, expression of CXCL12/SDF-1 mediates the interaction of BMSCs with BM endothelial cells in order to contribute to the maturation of megakaryocytes and thrombopoiesis. SDF-1 also initiates trans-endothelial migration of BMSCs in homing process via activation of integrins (LFA-1, VLA-4, and VLA-5) (90, 91). Moreover, BMSC secretory products, including leukemia inhibitory factor (LIF), macrophage stimulating factor (MIF), granulocyte-colony stimulating factor (G-CSF), OPN, IL-6, tumor necrosis alpha (TNFα) affect immune cell behavior (102, 107, 108). Costa et al. showed that osteoblast-derived lipocalin 2 (LCN2), with its anti-senescent function, regulates HSC progenitors and their proliferation capacity (106). Functional studies indicate that thrombopoietin and angiopoietin secreted by osteoblasts promote HSC quiescence (94, 98, 109), while CXCL12 regulates HSC migration in BM (110, 111). In addition, osteoblasts may regulate the activity of osteoclasts (derived from myeloid precursors in BM) in order to attract them to the site of resorption, thereby maintaining bone homeostasis (112). Osteoblasts produce receptor activator of nuclear factor-κβ ligand (RANKL) and osteoprotegerin (OPG), two critical factors in osteoclast differentiation and activation (103). Osteocytes (mature osteoblasts) modulate myelopoiesis via activation of Gsα-dependent signaling, which regulates secretion of G-CSF (100, 113). These data point out the importance of maintaining BM homeostasis, which is based on the molecular interactions among different cell types present in BM. And changes in local BM microenvironment induced by metabolic status of organism may shift this balance in favor of action of specific progenitors, which disrupt the priming of immune cell progenitors arising in BM in their function when they reach circulation.

**Table 2:** Secretory factors of BMSCs contributing to BM microenvironment and bone homeostasis.

**Secretory factors**	**Function**	**Obesity**	**Life-style interventions**
PGE_2_	Anti-inflammatory, inhibition of T cell proliferation (72)	↓ (59)	Omega 3 dietary intervention ↓ (82)
HGF	Anti-inflammatory, inhibition of T cell proliferation (72)	–	–
TGFβ	Anti-inflammatory, inhibition of T cell proliferation (72)	↓ (66)	–
IL-7	B cell development (83)	↓ (84)	–
IL-15	T cell homeostasis (85)	↓ (86)	–
IL-21	NKT cells maturation (87)	–	–
TNFα	HSC proliferation and activation (88)	↓ (60), ↑ (89)	–
CXCL12/SDF-1	Stem cell migration (90, 91)	↓ (92)	↑ Exercise, caloric restriction (68, 93)
Thrombopoietin	HSC quiescence (94, 95)	↑ (96)	↓ thrombopoiesis (97)
Angiopoietin	HSC quiescence (98)	↑ (99)	–
M-CSF	Myelopoiesis (100)	= (101)	–
G-CSF	Myelopoiesis (100)	↑-(101, 102)	↑ Sleeve gastrectomy (100)
RANKL	Osteoclast differentiation (103)	↑ (104)	= Exercise (105)
OPG	Osteoclast differentiation (103)	↓ (104)	= Exercise (105)
LCN2	HSC proliferation, inhibition of senescence (106)	↓ (60)	–

## Secretory Factors of Hematopoietic Stem Cells Affecting Bone Homeostasis and Immune Cell Properties

Multipotent HSCs represent another cellular component of BM, which are recognized as the ancestors of blood cells (114–116). Traditionally, HSCs differentiate into myeloid lineage (e.g., erythrocytes, granulocytes, macrophages, monocytes, and platelets) or lymphoid lineage [e.g., B lymphocytes, T lymphocytes, and natural killer (NK) cells] (117). While myeloid cells mature in the BM, human lymphoid cells must migrate to other lymphoid organs (e.g., thymus) in order to complete their maturation. In most of the experimental models, multipotency of HSCs is coupled with self-renewal abilities (118). HSCs together with endothelial cells (119), LepR^+^ stromal cells (120), megakaryocytes (121), sympathetic nerves, non-myelinating Schwan cells (122) and secreted bioactive molecules (123–126) create a dynamic BM microenvironment (127). Table 3 summarizes HSC secretory factors contributing to BM homeostasis along with their functions and changes in metabolic complications and life-style interventions. The BM microenvironment mediates signals for HSCs to differentiate into particular cell type in response to infection or blood cell destruction (142, 143). Interactions between HSCs and BMSCs are tightly interlinked by secreted signals and regulatory factors affecting the quiescence, self-renewal or mobilization of stem cells. HSCs are capable of receiving and producing signals that directly dialogue with the immune system. A recent study by Mitroulis et al. identified developmental endothelial locus-1 (Del-1) glycoprotein secreted by several components of HSC niche such as endothelial cells, reticular cells as a regulator of long-term HSC proliferation and differentiation toward the myeloid lineage (139). Another protein expressed in extracellular matrix of BM is tenascine-C (TN-C), important for active bone remodeling and HSC renewal in the endosteal region in conditions of hematopoietic stress (140, 144, 145).

**Table 3:** Secretory factors of HSCs contributing to BM microenvironment and bone homeostasis.

**Secretory factors**	**Function**	**Obesity**	**Life-style interventions**
IL-1β	HSC activation (128)	↓ (60)	↓ Dietary restriction- reduced intake of amino acids (129)
MCP1	HSC activation (128)	↓ (60)	–
TNFα	HSC activation (128, 130)	= (60)	–
Wnt10b	Bone formation (131)	↓ (132)	–
CXCL16	Osteoblast migration (133)	–	–
TRAP	Osteoclast activation (134)	= (60) ↑(135)	↓ Caloric restriction (136)
LIF	Osteoblast migration (133)	–	–
CTSK	Collagen degradation (137)	–	–
CTHRC1	Bone formation (138)	–	–
Del-1	HSC proliferation and differentiation (139)	–	–
TN-C	Bone remodeling and bone renewal (140)	↑ (141)	–

In the context of inflammation, HSCs are recognized as primary responders to infection, and the secretion of pro-inflammatory cytokines during infection is important for HSC regulation. This cascade of pro-inflammatory cytokines and signaling molecules includes IL-1, IL-2, IL-8, TLR4 (146), TNFα (147), IFNα, β, and γ (148) to activate T cells, NKT, and IL-4 and IL-6 to activate B cells [reviewed in King and Goodell (149)]. These cytokines are required for the maintenance of the appropriate proliferation and differentiation of HSCs in the steady-state and stress-induced condition.

Another cell type derived from the myeloid lineage are osteoclasts (“bone macrophages”), which are key players in process of bone resorption. Osteoclasts are specialized multinucleated cells derived from monocyte fusion containing from 2 to 12 nuclei per cell (150). The process of osteoclast differentiation is regulated via main activators of osteoclast formation, RANKL and M-CSF. In addition, RANKL promotes osteoclast resorption activity (151). In healthy conditions, osteoclasts play an important role in replacing of old or damaged bone matrix (bone resorption), which is followed by osteoblasts forming a new mineralized bone matrix (bone formation). This renewal process of bone matrix is also known as bone remodeling, which is energetically demanding (152). During this process, osteoclasts communicate with osteoblasts through cytokines such as TGF-β and IGF-1, which promote migration of BMSCs to newly resorbed tissue (153, 154). TGF-β can also induce expression of CXCL16, LIF, and Wnt10b by osteoclasts, which induce mineralization and recruitment of osteoclasts to osteoblasts (131, 133). Activated osteoclasts further produce secreted factors supporting their resorption activity, including cathepsin K (CTSK), sphingosine-1-phospate (137), tartrate-resistant acid phosphatase (TRAP) (134). CTSK is cysteine protease secreted by osteoclasts with an essential function in degradation of matrix collagen and activation of TRAP (155, 156). Mutation in CTSK leads to pycnodysostosis, rare autosomal recessive skeletal dysplasia, during which osteoclasts function is defected. Animal models with this deficiency showed reduced bone resorption, which together with normal or increased bone formation led to osteopetrotic phenotype (137). TRAP is a phosphatase expressed by osteoclasts and macrophages participating in skeletal development, collagen synthesis, and degradation or mineralization of bone matrix (134). Another molecule secreted by osteoclasts is collagen triple helix repeat containing 1 (CTHRC1), which serves as a positive regulator of osteoblastic bone formation (138, 157). These data provide further evidence that HSCs are capable of producing several inflammatory molecules, which contribute to creation of the BM microenvironment. Importantly, HSC differentiation in process of building active immune cells is under control of several bioactive molecules and signaling pathways, which need to be tightly regulated in response to metabolic or inflammatory stressors.

## Obesity-Induced Changes in Bone Marrow

Obesity is characterized by low-grade inflammation, challenging the immune cell responses in peripheral tissues. Further, the obesogenic condition increases BM cellularity 20–30% (101), changes BM composition of HSC and BMSC subpopulations and affects their differentiation capacity and increases white and red blood cell counts (Table 4) (96, 158, 164, 165, 172). Conditions associated with metabolic dysregulations, including hyperglycemia and hypercholesterolemia, have been linked to hematopoietic disruption and particularly to myeloid skewing (84, 165, 183).

**Table 4:** The changes in cellular composition of hematopoietic stem cells and bone marrow mesenchymal stem cells in bone marrow in obesity, exercise and dietary interventions.

**Cell type**	**Obesity**	**Exercise**	**Dietary intervention**
Erythrocytes	↑ (158)	↓ (159)	↑ (160) ↓ (161)
Lymphocytes	↓ (162)	↑ (162, 163)	↑ (160)
Monocytes (Osteoclasts)	↑ (164–166)	↓ (167)	↓ (167)
Eosinophils	↓ (168, 169)	–	↑ (169)
Basophils	↑ (170)	↑ (171)	–
Neutrophils	↑ (164, 165, 172)	↓ (173, 174)	↓ (174)
Thrombocytes	↑ (96)	–	↓ (97)
Chondrocytes	↓ (175, 176)	↑ (177)	↑ (178)
Osteoblasts	↓(60)	↑ (162, 163)	↑ (179, 180)
Bone marrow adipocytes	↑(60)	↓ (162, 163)	↓ (181, 182)

Hyperglycemia drives myelopoiesis and activation of neutrophils in the BM of obese mice (164, 165). Moreover, HFD-induced changes in bone architecture and immune cell homeostasis showed bone loss and a shift of HSC differentiation in myeloid over lymphoid progenitors (60, 162, 184). Further, morbid obesity elevated neutrophils in circulation and primed their immune function and metabolic activity, suggesting a higher inflammatory response in obesity-related diseases associated with impaired whole-body glucose metabolism (172). Another study by Kraakman et al. demonstrated that an obesogenic condition coupled with high glucose levels promotes increased thrombopoiesis via interaction of neutrophil-derived S100 calcium-binding proteins A8/A9 (S100A8/A9) and thrombopoietin in hepatocytes, which in turn leads to megakaryocyte activation and thrombocyte maturation in BM (96). Also, eosinophils with their anti-inflammatory activity have been shown to be affected by obesity, evidenced by decreased accumulation in AT and enhanced trafficking from BM to lung during allergic asthma (168, 185). Obesity-induced changes have been attributed also to basophils, which participate in lung inflammation and allergic reaction associated with metabolic complications (170).

It has been shown that differentiation capacity of BMSCs is changed by obesity in favor of increased adipocyte differentiation and impaired osteoblast and chondrocyte differentiation, which contributes to impairment of bone homeostasis and production of secretory factors affecting the function of neighboring cells in BM (60, 175, 176, 186). Liu et al. (54) recently reported an impairment of BMSC mobilization and selective migration of specific immune cells from BM into circulation in obesity. Further, Ferraro et al. showed a negative effect of diabetes on HSC mobilization capacity by changing the BM microenvironment (92). Not only proportion of immune cells in BM, but also secretion of inflammatory cytokines is modified by obesity (see some examples in Table 2). For instance IL-15 with its anti-obesity effect, TGF-β and IL-7 with their immunosuppressive properties are decreased with obesity in BM (66, 84, 86).

Previous studies in rodents under HFD condition have demonstrated increased pro-inflammatory BM microenvironment (e.g., TNFα, IL-6, and IL-1β) measured in BM or bone lysates (89, 104, 187). Our recent publications have reported that obesity does not induce increased inflammatory responses in BMSCs and HSCs of HFD mice or obese individuals compared to lean, which is accompanied with no change or decrease in osteoclast resorption activity (60, 188). This finding was also found in the study by Trotter et al., showing no changes in the mRNA levels of inflammatory markers in BM of HFD mice compared to lean (101). Further, obesity was identified as a negative factor of bone homeostasis in relation to osteoclast formation (104, 166, 189). Halade et al., using 12 months old female mice fed with 10% corn oil as a model of age-associated obesity, showed that increased adiposity enhances pro-inflammatory cytokine production (e.g., IL-1β, IL-6, and TNFα) and was associated with a higher differentiation of osteoclasts (104, 190). Another animal study using 5 weeks old male mice found higher rates of osteoclast precursors, as well as elevated osteoclast formation, bone resorption activity and increased expression of RANKL, TNFα, and TRAP (166). In addition, acute exposure to dietary fatty acids increased osteoclastogenic activity in circulating monocytes and increased secretion of cytokines (191). However, this study did not investigate the osteoclast in BM and their resorption activity. In our animal study using a HFD model (60% calories from fat) in 12 weeks old C57BL/6 male mice, we did not observe any significant changes in osteoclast activity or number (60). In clinical study (188) examining obese subjects, we found decreased bone resorption and bone formation activity, suggesting a slowing of bone turnover. The discrepancies between studies may be explained by using different animal models, length/composition of the diet, or different source of bone cells for measurement of inflammatory condition in BM.

In terms of HSC secreted molecules (e.g., CXCL16, CTSK, Del-1, LIF, or CTHRC1), which play an important role in bone homeostasis and metabolism (Table 3), there is very limited information about expression changes in the BM during obesity. Thus, these observations suggest that further studies are needed in order to investigate the inflammatory status of BM cells and their microenvironment in obesity in relation to bone and whole-body metabolism.

However, it raises further questions whether obesogenic condition activates immune cells in BM or immune cells need to migrate through the circulation into the target tissue, i.e., adipose tissue (AT), skeletal muscle, liver to activate their inflammatory status. This would suggest that BM is a primary site of immune cell production and plays an important role in immune cell mobilization into circulation, whereby these cells are directed to traffic into the sites of inflammation.

## Life-Style Interventions: Dietary and Physical Activity Interventions Improve Obesity-Induced Changes in Bone Marrow Homeostasis

In obesity and type 2 diabetes, several approaches have been applied to treat or prevent the detrimental effects of metabolic complications. These include physical activity, as well as dietary or pharmacological treatment. As a lot of investigations have been focused on the metabolic and inflammatory improvements in peripheral tissues, there is limited information on these parameters in relation to BM homeostasis (Tables 1–4).

Dietary supplementation with long-chain n-3 (ω-3) PUFAs, supplied as fish oil (FO), which is known for its anti-inflammatory effects, demonstrated to be beneficial for skeletal health, as evidenced by increased osteogenesis and decreased osteoclastogenesis (179, 180). A recent study by Cao et al. (192) reported that 6 months of a FO diet increased bone density and microstructure. However, they did not investigate bone adiposity or inflammatory responses in BM in these conditions.

Exercise also showed a positive effect on bone homeostasis. In rodents and humans, exercise has been shown to increase bone density, decrease bone adiposity, and improve chondrogenesis in HFD mice and humans (177, 193, 194). Further, increased physical activity has been shown to promote HSC proliferation and differentiation and modulate immune cell composition in circulation (159, 171, 173, 195–197) (Table 3). An additional effect of exercise on the bone is increased mechanical stress for skeletal system induced by whole body vibration (WBV), which has been shown to improve bone density by reducing bone marrow adiposity in mice and humans and restoring lymphopoiesis (increased number of B cells). WBV also showed an effect on immune cells in circulation and induced lower infiltration in AT (162, 163). A key strategy to prevent obesity and its complications, including bone health, is a combination of exercise and a well-balanced diet. A study by Garbiax et al. using 11 months old male rats showed that exercise along with a caloric restricted diet (low fat and low sucrose) decreased bone resorption and osteoclast number in the obese state (167). However, the inflammatory properties of immune cells in BM also have not been investigated following these interventions.

The effects of caloric restriction on bone health is still poorly understood. Generally, caloric restriction, accompanied with weight loss, has a positive effect on systemic glucose tolerance and inflammatory status of immune cells and their count (neutrophils) (169, 174). A recent study by Collins et al. (160) showed that dietary caloric restriction protects BM and optimizes immunological responses of immune cells by enhanced accumulation of memory T lymphocytes in BM, erythropoiesis and bone marrow adiposity. However, caloric restriction or starvation in growing mice leads to increased accumulation of bone marrow fat even though peripheral adipose tissue (AT) mass is decreased. Further, it causes decreased bone density and increased bone resorption (161, 198, 199). Caloric restriction had a similar effect in patients with anorexia nervosa (200). However, in the obese condition, caloric restriction may have a positive effect, as evidenced by reduced bone adiposity and improvement of bone density and chondrogenesis. Although no results on inflammatory components in BM have been measured in this setting (178, 181, 182).

In the context of inflammatory cytokine production, a recent publication showed that reduced intake of amino acids may inhibit secretion of pro-inflammatory mediator IL-1β mediated by myeloid precursors in BM (See also examples in Table 3) (129). However, for most of the above-mentioned inflammatory proteins (Table 3) (e.g., MCP1, TNFα, LIF, CTSK, CXCL16, CTHRC1, Del-1, or TN-C), there is a lack of information in the literature about the modulation of their secretory activity in dietary interventions in BM, which indicates that this area of research needs to be further investigated. Based on the recent publications in relation to life-style interventions and bone health, it suggests that further studies are needed to dissect the role of inflammatory components in BM homeostasis and how these may contribute to local BM and systemic metabolic regulation.

## Conclusions and Perspectives

BM is an important immune organ, whereby immune and progenitor cells with different functions interact with each other and affect local and systemic immune conditions in response to metabolic and inflammatory stressors, including obesity. Obesity leads to a pro-inflammatory state, which influences metabolic function in insulin-responsive tissues including bone and its immune compartment, BM. Further, the obesogenic condition induces BM hyperplasia defined by increased number of immune cells (monocytes, neutrophils, thrombocytes etc.) migrating into the circulation, which are usually primed in higher inflammatory responses to activate inflammation in peripheral tissues.

How can we define an inflammation in BM? Is it a process of bone resorption defined by activation and expansion of osteoclasts in BM or a process accompanied by increased secretion of inflammatory cytokines, which we know from definition of inflammation in peripheral tissues? And how is it affected in metabolic complications? Most studies have reported changes on the level of osteoclast resorption activity, but not much on secretory properties of immune cells in BM niche. Another aspect of inflammatory status in BM is the immunosuppressive properties of BMSCs, which also contribute to immune regulation in BM microenvironment through cell to cell interactions and secretory bioactive molecules to maintain BM homeostasis.

**Figure 2:**
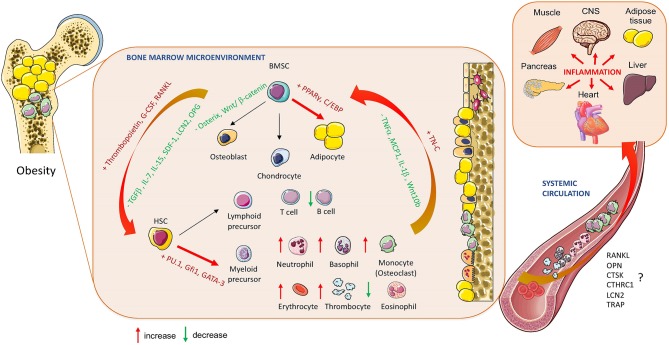
Obesity-induced changes in bone marrow homeostasis. The effect of obesity on BM cellular composition and secretory profile of bioactive molecules produced by hematopoietic stem cells (HSCs) and bone marrow mesenchymal stem cells (BMSCs) in relation to systemic changes affecting whole-body metabolism and inflammation (RANKL, Receptor activator of nuclear factor-κβ ligand; OPN, Osteopontin; CTSK, Cathepsin K; CTHRC1, Collagen triple helix repeat containing 1; LCN2, lipocalin 2; TRAP, Tartrate-resistant acid phosphatase; OPG, Oteoprotegerin; TGF-ß, Transforming growth factor beta; IL-7, Interleukin 7; IL:15, Interleukin 15; IL1-ß, Interleukin 1 beta; SDF-1, Stromal cell derived factor; TNFα, Tumor necrosis factor alpha; MCP1, Monocyte chemoattractant protein 1; TN-C, Tenascine C; PPARγ, peroxisome proliferated-activated receptor gamma; C/EBP, CAAT enhancer binding protein; Gfi1, Zinc finger protein Gfi1; GATA3, GATA binding protein 2). Cell animations were adapted from SERVIER Medical Art; https://smart.servier.com to create the figure.

Undoubtedly, metabolic stressors such as obesity interrupt the existing balance among BMSC and HSC functions, which further affect systemic whole-body immune regulation in relation to metabolic status of organism. Many bone cell-secreted molecules have been found to play an important role in the regulation of AT development (e.g., RANKL, CTSK, and CTHRC1) (201–203) (Figure 2). Therefore, studying their function in relation to bone and fat metabolism is of interest. However, more studies are needed to understand the role of inflammatory changes and crosstalk between immune cells and BMSCs in BM in response to obesity and how these changes can be modulated with targeted therapies focused on treatment for bone and metabolic complications.

## Author Contributions

AB and MT researched data and wrote the manuscript. MT reviewed and edited the manuscript.

## Conflict of Interest

The authors declare that the research was conducted in the absence of any commercial or financial relationships that could be construed as a potential conflict of interest.

## Abbreviations

AT: Adipose tissue
BATF: Basic leucine zipper transcription factor, ATF-like
BM: Bone marrow
BMI1: B lymphoma Mo-MLV insertion region 1 homolog
BMP: Bone morphogenic protein
BMSC: Bone marrow mesenchymal stem cell
C/EBP: CAAT enhancer binding protein
CD25 (CD80, CD86, CD40, CD69): Cluster of differentiation 25, 80, 86, 40, 69
Cdc42: Cell division control protein 42 homolog
CTSK: Cathepsin K
CTHRC1: Collagen triple helix repeat containing 1
CXCL12: C-X-C motif chemokine 12
Del-1: Developmental endothelial locus 1
DNMT1: DNA methyltransferase 1
FO: Fish oil
GATA: 1-3- GATA-binding protein 1-3
G-CSF: Granulocyte–colony stimulating factor
GFI1-: Zinc-finger protein GFI1
H4K15ac: Acetylation on lysin 16 of histone 4
HGF: Hepatocyte growth factor
HSC: Hematopoietic stem cell
IDO, Indoleamine 2: 3-dioxygenase
IFNα: Interferon alpha
IFNβ: Interferon beta
IFNγ: Interferon gama
IGF-1: Insulin-like growth factor 1
IL-1 (IL-1β, IL-2, IL-4, IL-6, IL-7, IL-8, IL-15, IL-21): Interleukin 1-21
LCN2: Lipocalin 2
LepR: Leptin receptor
LFA-1: Lymphocyte function-associated antigen 1
LIF: Leukemia inhibitory factor
MCP1: Monocyte chemoattractant protein 1
MIF: Macrophage stimulating factor
M-CSF: Macrophage colony-stimulating factor
NKT: Natural killer cell
OPG: Osteoprotegerin
OPN: Osteopontin
PGE_2_: Prostaglandin E_2_
PPARγ: Peroxisome proliferator-activated receptor gama
PUFA–: Poly unsaturated fatty acid
RANKL: Receptor activator of nuclear factor-κβ ligand
RUNX2: Runt-related transcriptional factor 2
S100A8/A9: Neutrophil-derived S100 calcium-binding proteins A8/A9
SCF: Stem cell factor
SDF-1: Stromal cell-derived factor 1
sFRP1: secreted Frizzled-related protein 1
TGF-β_1_: Transforming growth factor beta 1
TLR4: Toll-like receptor 4
TN-C: Tenascine-C
TNFα: Tumor necrosis factor alpha
TNFβ: Tumor necrosis factor beta
TRAP: Tartrate-resistant acid phosphatase
Treg: Regulatory T cell
VLA-4 (VLA-5, VLA-7): Very late antigen 4, 5, 7
WBV: Whole body vibration
Zfp432: Zinc finger protein 432
Zfp521-: Zinc finger protein 521.
